# Iatrogenic chylous collection post laparoscopic nephrectomy

**DOI:** 10.1259/bjrcr.20190058

**Published:** 2020-09-29

**Authors:** Haseeba Tawfeeq, Su W Lim, Snehal Lapsia, Shofiq Al-Islam

**Affiliations:** 1 Royal Blackburn Hospital, Blackburn, England

## Abstract

With the increasing number of laparoscopic nephrectomies, trauma to lymphatic channels has become an increasingly recognised complication. Early diagnosis and prompt management are key to avoid highly morbid sequelae including severe malnutrition and immunodeficiency. This case reviews the important complication of a retroperitoneal chylous collection following laparoscopic radical nephrectomy.

## Case presentation

A previously healthy 71-year-old male presented to primary care with 1-week history of chest symptoms while on a holiday overseas. A CT pulmonary angiography (CTPA) was done; which confirmed the clinical suspicion of a pulmonary embolism. The inferior limit of the CTPA demonstrated a suspicious left renal mass. A subsequent CT abdomen and pelvis with contrast was arranged that showed 7.4 × 6.3 cm malignant mass thought to be a renal cell carcinoma in the left kidney. Left-sided radical nephrectomy was performed utilising an anterior retroperitoneal approach; with the removal of the left kidney, surrounding fat and local retroperitoneal lymph node dissection. The patient represented with back pain and fever 2 weeks post-operatively. A post-operative CT abdomen and pelvis with contrast demonstrated a large (approximately 1080 ml) left retroperitoneal collection indicating a working diagnosis of a post-operative abscess.

## Investigations/imaging findings

**Figure 1.**
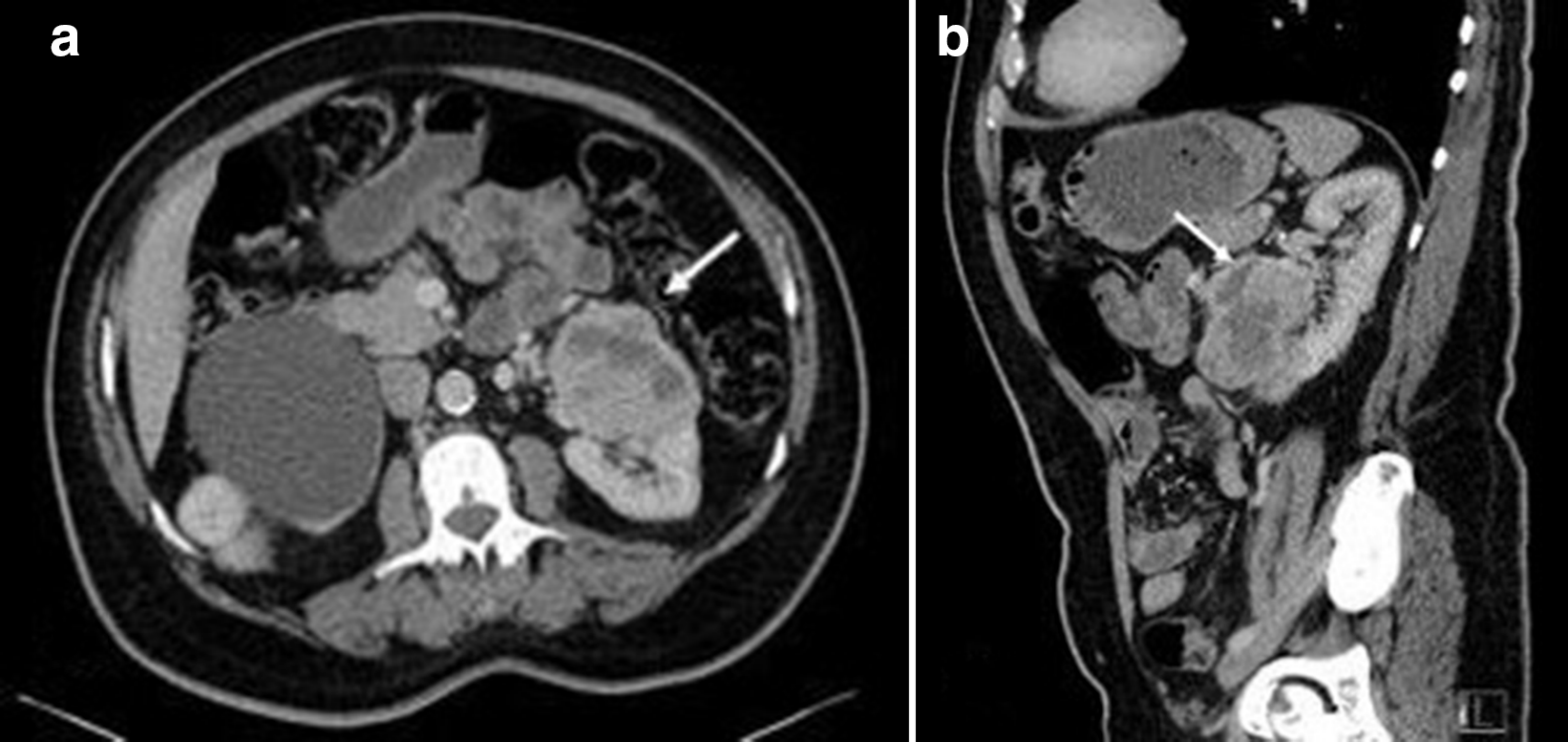
Axial (1a) and sagittal (1b) slices of CT abdomen and pelvis demonstrating the large renal mass in the lower pole of left kidney.

Following the patient’s acute presentation of a pulmonary embolism and the incidental finding of a suspicious looking left renal mass, a staging CT scan was organised. The chest, abdomen and pelvis were imaged in the portal venous phase. An 8 × 5.5 cm left renal mass was demonstrated with no size-significant nodal involvement (Figure 1). Histological results of the excised kidney and lymph node confirmed evidence of clear cell renal cell carcinoma with no nodal involvement.

**Figure 2.**
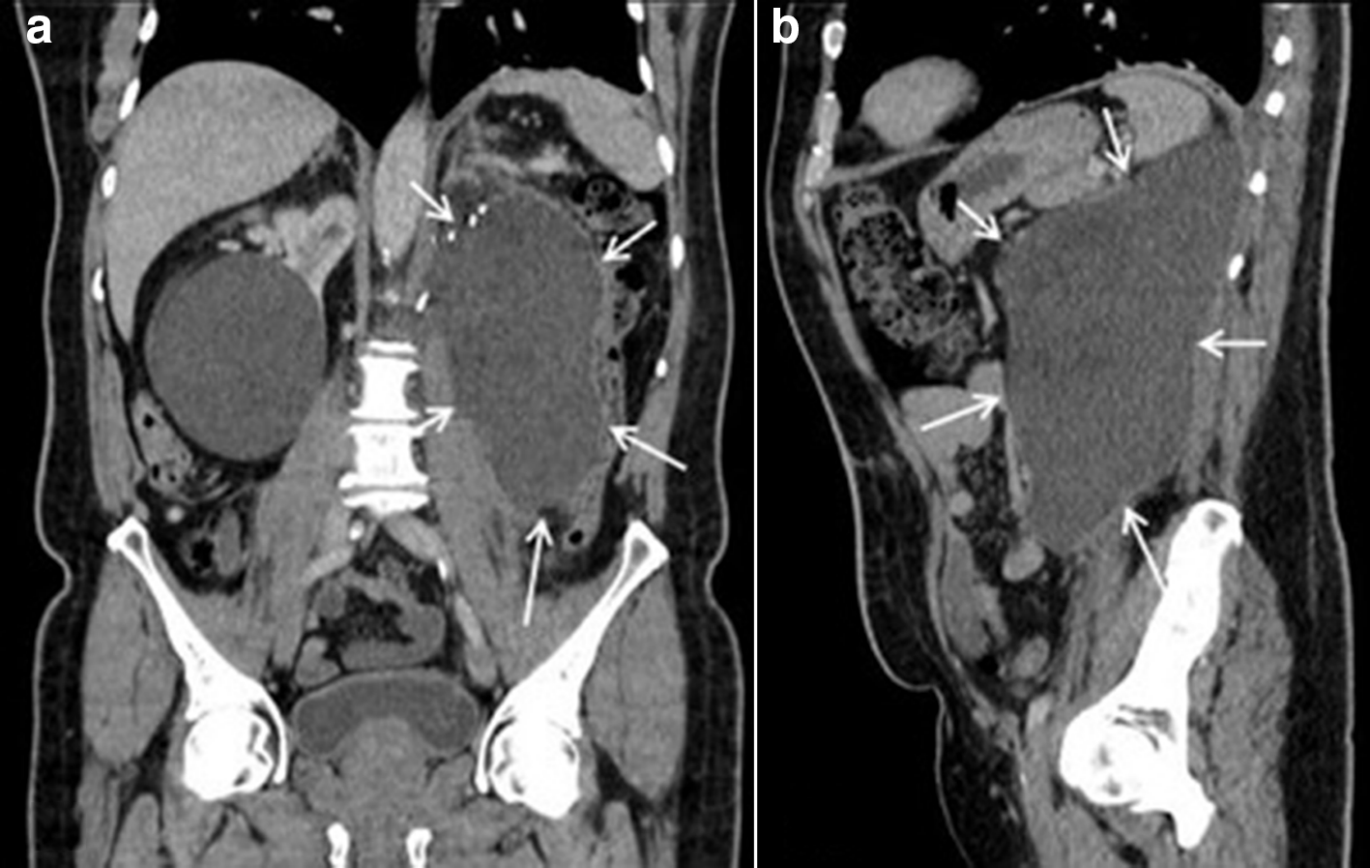
Coronal (2a) and sagittal (2b) slices of the CT abdomen and pelvis showing large collection in the left retroperitoneal region with varying density.

The patient had an uneventful recovery post-surgery and was discharged from the hospital 2 days later. 2 weeks after, the patient represented to emergency services with acute back pain and fever. Blood test showed raised CRP of 127 mg l^−1^ with a normal white cell count of 7.9 × 10*9/L. A repeat CT abdomen and pelvis with contrast was performed. Images of the abdomen in the portal venous phase were obtained and demonstrated a large left retroperitoneal collection with enhancing walls and minimal internal density variation (Figure 2). The initial diagnosis was of a post-operative abscess. The patient was commenced on Co-amoxiclav antimicrobial therapy. No clinical improvement was observed over 48 h, and the patient was referred to Interventional Radiology for drainage of the collection.

## Differential diagnosis

1. Post-operative abscess
2. Seroma
3. Haematoma
4. Chylous post-operative collection

## Treatment

**Figure 3.**
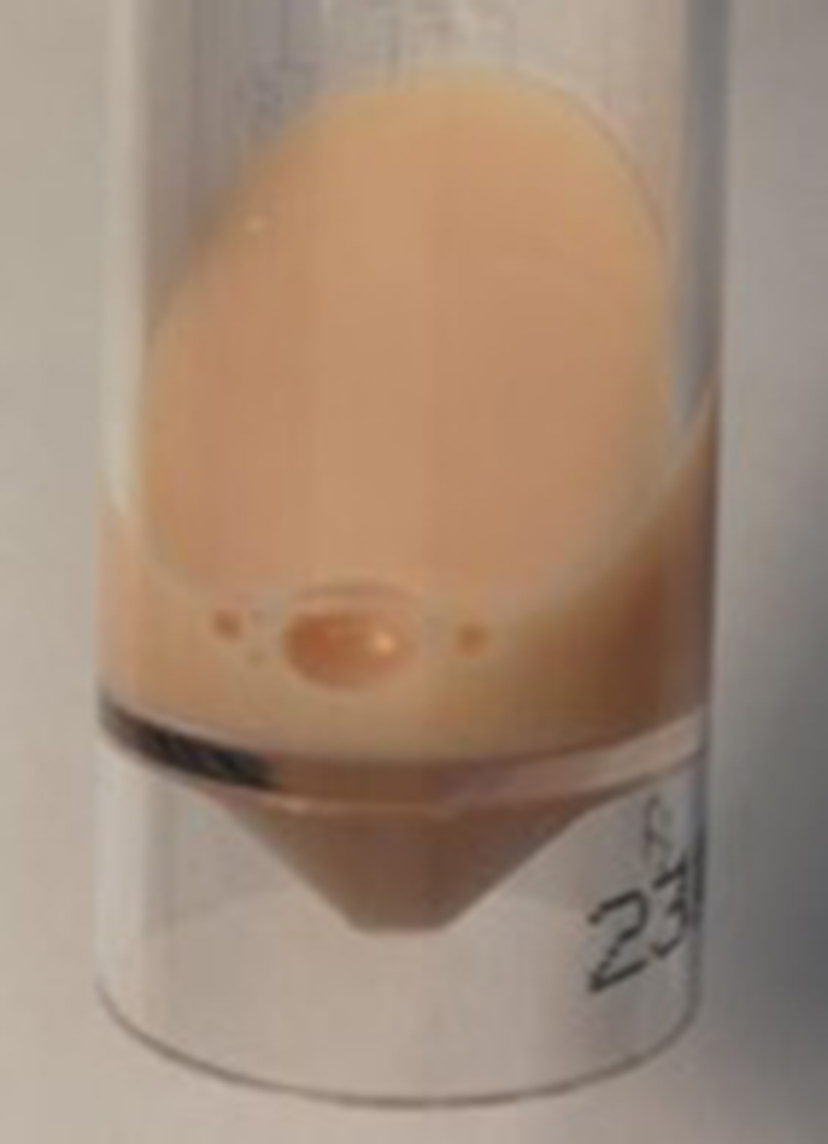
Sample of the retroperitoneal collection showing milky fluid.

**Figure 4.**
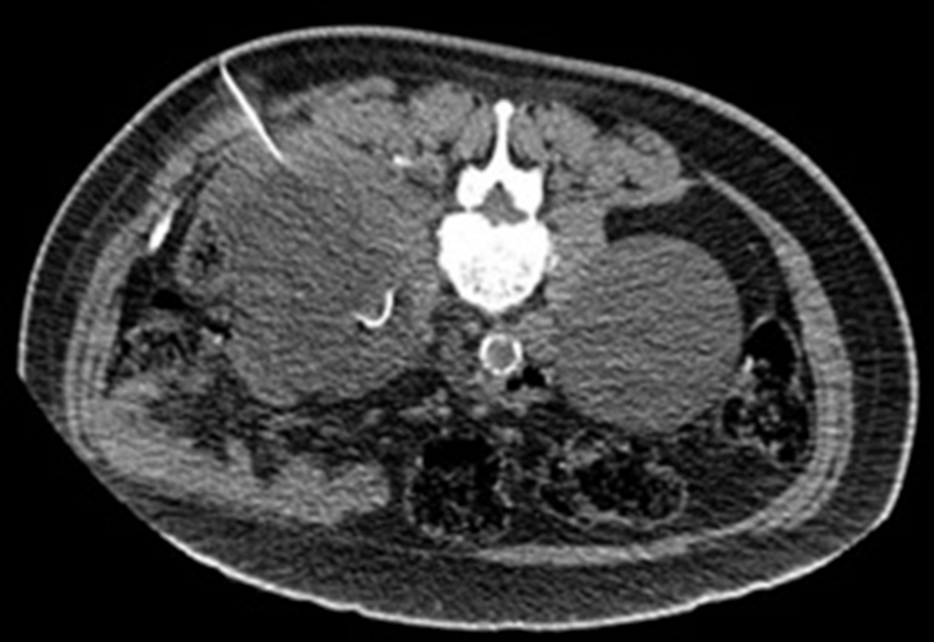
Axial slice of the non-contrast CT abdomen demonstrating the pigtail catheter within the left retroperitoneal collection.

Under direct CT fluoroscopy an access needle was introduced into the collection with the patient in a prone position. A 12 Fr pigtail locking catheter was inserted using Seldinger technique over a short stiff wire after serial dilatation (Figure 3). Approximately, 100 ml of non-odourous milky fluid was drained raising the possibility of chylous collection (Figure 4). Biochemistry established raised triglycerides levels within the sample confirming the suspicion of a chylous collection.

**Figure 5.**
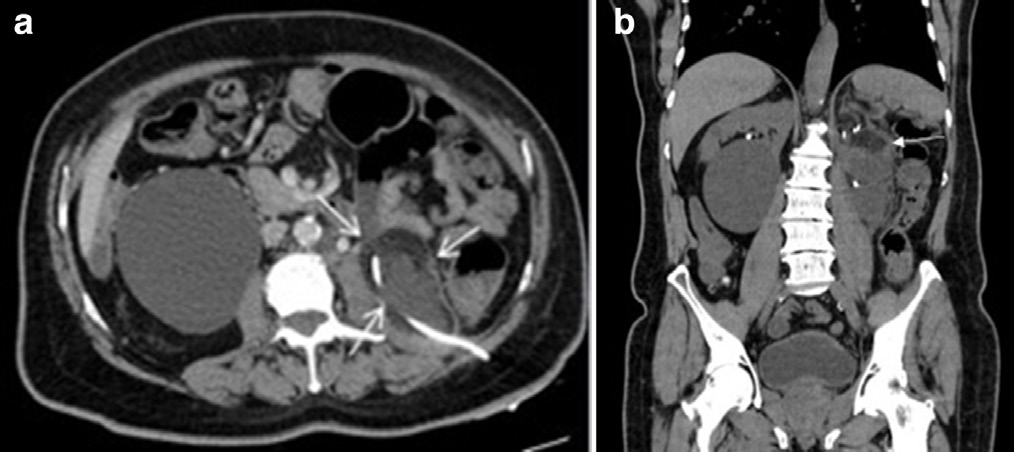
Axial and coronal slices of the CT abdomen and pelvis showing reduction in size of the left retroperitoneal collection with the drain still *in situ*. Fat–fluid level is best seen within the collection on coronal slice.

Advice was issued to the clinical team to monitor the drain output and the patient was started on total parenteral nutrition (TPN) to restrict his dietary intake of fat. Follow-up CT was performed which showed partial resolution of the collection following the insertion of the drain, with clear fat–fluid level within the collection; which is a pathognomonic feature of chyle on CT (Figure 5).

## Discussion

Iatrogenic injury to lymphatic channels is a recognised albeit rare post-operative complicatio^1^ It has become more prevalent due to the increased number of laparoscopic operations compared to the classic open surgery approach; which entailed better visibility and direct ligation of the lymphatic channels..^2^ Since the advent of laparoscopic practice, surgical outcome and recovery times have demonstrated its multitude of merits. However, awareness of the constraints of the operating field of view associated with laparoscopy is key to the prevention of lymphatic trauma associated complications.

Lymph formed in the kidney drains from the hilum through small lymphatics, into the extensive network of the retroperitoneal space then cyterna chyli and lastly the thoracic duct where the lymphatic system enters the systemic circulation via the left subclavian vein. The retroperitoneal lymphatics represent the primary draining sites of renal lymph and extend between the first and the fifth lumbar vertebrae.^3^ Thus, chylous injury is a recognised complication of radical nephrectomies which involves retroperitoneal lymph node dissection.

A review of the available literature suggests an average of 4.1 post-operative days is usually the time needed for a chylous collection to form when normal dietary intake is commenced.^2^ Intraabdominal pooling of chyle may be clinically indistinguishable from other types of post-operative collections on imaging. Symptoms can be attributed to other differentials and include abdominal pain, distension with or without a febrile illness. Patients may also complain of other symptoms such as nausea, vomiting, shortness of breath or surgical wound oozing.^4^

Diagnosis of chylous contents is usually suggested by examining the appearance of the leaking fluid. This is particularly useful if the patient has an indwelling catheter at the operative site. The colour and consistency of the drained fluid can be directly observed, and a sample can be biochemically tested for chylomicrons or triglycerides to confirm high fat levels.^5^ Since this is rarely the case, medical imaging is often performed first; of which, CT scan remains the modality of choice. However, the lack of CT findings specific for chylous fluid makes it a non-specific diagnostic marker for lymphatic contents. The only pathognomonic feature of chylous collection on CT scan is the presence of a “fat–fluid level,” (Figure 5) which is rarely shown at the early stages of the collection.^1^

Kim et al^6^ and Capocasale et al^7^ conducted in-depth studies of chylous leakage after laparoscopic nephrectomies. The vast majority of cases they reported underwent spontaneous resolution and did not require invasive intervention. The true incidence of lymphatic trauma and or chylous collection may never be fully appreciated as the majority of cases resolve without symptoms and thus are not investigated further.^2^

The aim of management of chylous collection is symptom relief and replacement of nutritional losses.^2^ As a result, high index of suspicion is key in recognising intra-abdominal chylous fluid and maximising the chances of conservative measures being successful. Often (similarly to this case reviewed) the chylous collection is not suspected timely and other treatment options are initiated like antimicrobial therapy for infective causes. Delays in considering lymphatic injury and chylous accumulation can lead to failure of conservative measures resulting in further morbidity. The initial intervention to consider is drainage of the collection percutaneously, usually under imaging guidance. Drainage enables removal of a potential for infection and allows the injured lymphatics to heal. Drainage alone is rarely sufficient and is combined with dietary modification including low-fat and high-protein diet, the use of somatostatin analogues and occasionally, total parenteral nutrition may be necessary in refractory cases.^2^ Conservative management is more likely to be successful with early diagnosis and dietary modifications. This would subsequently reduce morbidity; hospital stay lengths and lead to better management outcomes. Very few cases fail to respond to the aforementioned measures and require direct surgical ligation of the leaking vessel.^6^

As the practice of laparoscopic nephrectomy has gained a foot hold, the associated limitations became more prevalent. A number of case reports studied the presentation, diagnosis and response to treatment of chylous leakage after laparoscopic nephrectomies^2,3,6,7^. Due to its reported rarity, lymphatic injury is still an overlooked differential potentially leading to considerable diagnostic and therapeutic delay. Although most cases resolve spontaneously, this case emphasises the importance of early consideration of chylous leakage in the differential diagnosis of patients presenting with intra-abdominal collections following laparoscopic renal surgery.

## Learning points

- Chylous leakage resulting from lymphatic injury has become more prevalent with laparoscopic nephrectomies.
- Most cases respond well to conservative management which involves a multidisciplinary approach. The aim of which is to reduce lymphatic flow to the damaged channels, reducing risk of infection, optimising nutritional requirements along with providing symptomatic relief.
- Conservative treatment is more successful with early diagnosis and prompt treatment depending on the severity of the chylous leakage.
- Image-guided minimally invasive drainage of chylous collections is a feasible, safe and effective option offering less recovery time and less morbidity risk compared to re-look surgery.
